# Added value of the pulmonary vein pulsatility index and its correlation to neonatal umbilical artery pH in fetal growth restrictions: a Vietnamese matched cohort study

**DOI:** 10.1186/s12884-023-05910-0

**Published:** 2023-08-30

**Authors:** Minh Son Pham, Dinh Vinh Tran, Chi Kong Pham, Thi Linh Giang Truong, Vu Quoc Huy Nguyen

**Affiliations:** 1 Department of Prenatal Diagnosis, Da Nang Hospital for Women and Children, 402 Le Van Hien, Da Nang, Vietnam; 2 Department of Obstetrics and Gynecology, Hue University of Medicine and Pharmacy, 6 Ngo Quyen, 491200 Hue, Vietnam

**Keywords:** Pulmonary vein pulsatility index, Fetal growth restriction, Umbilical artery pH, Umbilical artery pulsatility index

## Abstract

**Background:**

In clinical obstetrics, many guidelines recommended the use of Doppler fetal ductus venosus blood flow to monitor and to manage fetal growth restriction (FGR). The ductus venosus and the pulmonary venous flow pattern of fetuses are similar. Umbilical artery pH (UA pH) is essential in identifying adverse pregnancy outcomes, particularly in fetal growth restriction cases. Nevertheless, the literature indicates that the relationship between pulmonary vein pulsatility index (PVPI) and UA pH in FGR cases has not been well investigated. This study aimed to identify the alteration in PVPI in FGR cases and evaluate the correlation between PVPI and UA pH in FGR newborns.

**Methods:**

This matched cohort study of singleton pregnancies from 28^+ 0^ to 40^+ 0^ weeks of gestation without congenital abnormalities included 135 cases of FGR (disease group) and 135 cases of normal growth (control group). The PVPI was measured at the proximal segment of the right or left pulmonary vein, approximately 5 mm from the left atrium wall. The umbilical artery pulsatility index (UAPI) was measured on the free umbilical cord. An elective cesarean section or labor induction are both options for ending the pregnancy, depending on the condition of the mother or fetus. Umbilical artery blood samples were collected within 5 min of delivery for UA pH measurement. SPSS version 20 and Medcalc version 20.1 were used for data analysis.

**Results:**

FGR cases had a significantly higher mean fetal PVPI than the control group (1.16 ± 0.26 vs. 0.84 ± 0.16; p < 0.01), and PVPI and UAPI were positively correlated (r = 0.63; p < 0.001). PVPI and UA pH were negatively correlated in FGR patients, with r = -0.68; p < 0.001. The PVPI value on the 95th percentile had a prognostic value of UA pH < 7.20 with a sensitivity of 88.2%, specificity of 66.3%, positive predictive value of 46.9%, and negative predictive value of 94.3%.

**Conclusions:**

There was a statistically significant difference in PVPI values in FGR cases compared to the normal growth group, a positive correlation between PVPI and UAPI, and a negative correlation between PVPI and UA pH. PVPI might have a prognostic meaning in predicting UA pH at birth.

**Supplementary Information:**

The online version contains supplementary material available at 10.1186/s12884-023-05910-0.

## Introduction

With the development of color Doppler ultrasound, fetal vascular Doppler ultrasound has become increasingly important in clinical obstetric practice. Studies were done 20 years ago to establish the value of the normal fetus’s pulmonary artery and pulmonary vein pulse indices. Later research also created reference tables for various study populations [1, 2]. The reference range for fetal pulmonary venous Doppler values was established by the study by Bahlmann et al. on 365 singleton pregnancies with pregnant women of Caucasian origin [3]. An inverse relationship between the pulmonary venous pulsatility index and gestational age was found in a study of 168 singleton pregnancies in Vietnam that had proper fetal development and were between 28 and 40 weeks gestation when pulmonary vein Doppler measurements were taken [4]. Several studies have demonstrated that PVPI is a valuable criterion for predicting the outcome of surgical repair of left ventricular hypoplasia in the postpartum period [5]. PVPI could independently predict small for gestational age fetuses, and the efficacy was comparable to estimated fetal weight during pregnancy [6].

The Doppler indices measured from the ductus venosus, umbilical arteries, cerebro-placental, and umbilico-cerebral Doppler ratios have been shown to correlate with the well-being of fetal growth restriction, which includes umbilical artery blood pH [7–9]. Umbilical artery pH (UA pH) is essential for assessing adverse pregnancy outcomes since it predicts the risk that neurodevelopmental problems will manifest in childhood [10]. There is a redistribution of fetal circulation in situations of FGR, so the Doppler ductus venous flow is changed, ranging from an increased pulsatility index to the absence or reversal of the A wave. The pulmonary veins’ Doppler flow reflects pressure in the left atrium, and its Doppler waveform is identical to the ductus venosus [11]. As a result, the fetal pulmonary vein pulsatility index undergoes the same alterations as the ductus venosus in specific fetal conditions. Likewise, several investigations have shown that fetal growth restriction or placental insufficiency increases the PVPI [12, 13]. However, no studies have investigated the relationship between PVPI and UA pH in fetal growth restriction. The present study was conducted with two objectives: (1) to investigate the change in PVPI in FGR cases and (2) to evaluate the correlation between PVPI and UA pH in fetuses with FGR.

## Materials and methods

This matched, prospective cohort study was conducted at the Da Nang Hospital for Women and Children, Danang City, Vietnam, from April 2017 to December 2019. The selection criteria for participants were as follows:

- The group with FGR cases (disease group): gestational age between 28^+ 0^ and 40^+ 0^ weeks; no fetal morphological abnormalities in utero and also after birth; an umbilical artery pulsatility index above the 95th percentile or cerebro-placental Doppler ratio (CPR) below the 5th percentile for gestational age; and an estimation of fetal weight (EFW) and birth weight below the 10th percentile for gestational age.
- The group with fetal growth appropriate for gestational age (control group): gestational age between 28^+ 0^ and 40^+ 0^ weeks; no fetal morphological abnormalities in utero and also after birth; an umbilical artery pulsatility index below the 95th percentile for gestational age; estimation of fetal weight and birth weight in the range of the 10th percentile to the 90th percentile for gestational age.

The estimation of fetal weight was based on *Hadlock 4’s* formula. The newborn’s birth weight was compared to the *Hadlock 4* reference interval. The UAPI and CPR reference table was applied according to the recommendations of the New Zealand Maternal Fetal Medicine Network [14].

The sample size was calculated using the following formula to estimate the proportion of the population:

$${\rm{n}} \ge {\rm{Z}}_{1 - \alpha /2}^2\frac{{p\left( {1 - p} \right)}}{{{\Delta ^2}}}$$

where α = 0.05; using a 95% confidence level, the value of Z _1-α/2_ is 1.96, p = 7% (the rate of FGR), and ∆ = 0.05 (the expected deviation between the proportion obtained from the sample and the proportion of the population).

The minimum sample size calculated for the FGR group was 100. In this study, we enrolled 135 pregnant women with FGR. The normal fetal growth group also included 135 patients.

The fetuses were closely monitored following the local protocol when fetal growth restriction was revealed. Based on Doppler alterations in the ductus venous and umbilical artery, the fetuses were examined by ultrasound once or twice a week. The Doppler values of the ductus venous, umbilical artery, pulmonary venous, amniotic fluid, and EFW were measured on each examination. The cardiotocography was performed immediately after the obstetric ultrasound. The pregnancy was terminated when the mother had medical complications, fetal distress was detected, or the pregnancy reached the 38th week of gestation. Depending on the mother’s or fetus’s status, delivery was indicated either by elective cesarean section or induction of labor.

**Fig. 1:**
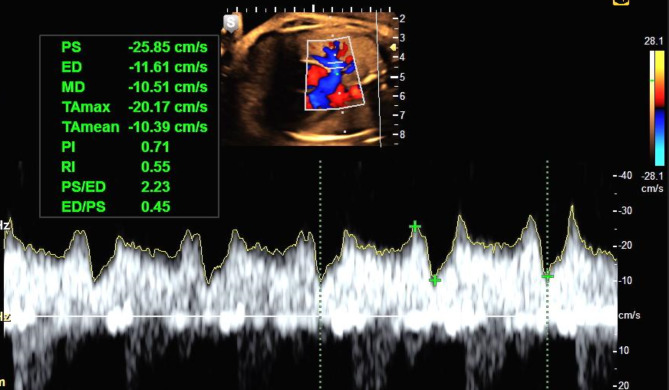
The measurement of the pulmonary vein Doppler and the Doppler waveform

A single doctor who qualified of the Fetal Medicine Foundation performed all ultrasound scans using a Voluson S6 ultrasound machine. UAPI was measured in the free umbilical cord. PVPI was measured at the proximal segment of the right or left pulmonary vein, approximately 5 mm from the left atrium wall. The 2–3 mm Doppler window and an ultrasound beam angle of less than 30° allowed measurements and angle correction to be performed if the ultrasound beam angle was greater than 30° (Fig. 1). Doppler index measurements were performed when the pregnant woman was lying still, the fetus had no breathing movement, and the fetal heart rate was regular and in the range of 120–160 beats/minute. After three measurements that meet the specified requirements, the UAPI and PVPI are averaged. Fetal Doppler indices were assessed within three days from delivery. The classification of PVPI was based on the PVPI value demonstrated in normal growth fetuses according to current research in Vietnam as described elsewhere [4]. The Doppler indices during the final prenatal assessment were obtained for analysis of the study’s outcomes.

Cardiotocography (CTG) was routinely performed in all patients before delivery. The CTG findings were obtained using a fetal monitor type BT-350. According to FIGO guidelines published in 2015, CTG results were divided into three groups: normal, suspected, and abnormal [15].

Umbilical artery blood samples were collected within 5 min of delivery and sent to the laboratory within 60 min for arterial blood gas analysis using GASTAT 1835. The skin-to-skin contact technique between the newborns and their mothers was subsequently performed. The UA pH is analyzed immediately after the blood sample is delivered to the laboratory.

Newborn babies were weighed, and their measurements were compared against the Hadlock-4 gestational age reference table. A pediatrician examined the newborns for abnormalities.

The adverse pregnancy outcome included: respiratory morbidity (tachypnea, neonatal respiratory distress, and respiratory support), an Apgar index < 7 at 5 min., neonatal intensive care unit (NICU) admission, and UA pH < 7.20.

Data analysis was performed using SPSS version 20 and Medcalc version 20.1. The algorithms employed in this process included percentage calculation, statistical estimation, and hypothesis testing, with a statistical significance of p < 0.5. The chi-squared test, Fisher’s test, or Yate correction were applied depending on the expected frequency in each data plot. Pearson’s correlation coefficient (r) was used to measure the correlation between the two variables. Analysis of sensitivity, specificity, positive likelihood ratio, negative likelihood ratio, positive predictive value, and negative predictive value was performed by entering the data into a 2 × 2 table. A multiple linear regression model is used to estimate the value of a dependent variable. The adjusted R-squared coefficient was evaluated to assess the fit of the regression model. The Durbin-Watson statistic is used to assess first-order series autocorrelation. The result does not contradict the first-order series correlation assumption if the Durbin - Watson value is between 1.5 and 2.5. ANOVA is performed to test the hypothesis regarding the model’s fit to the population (if sig. 0.05, the model fits the population). If the independent variable in the equation for the multiple linear regression model is more significant than 0.05, that variable does not affect the dependent variable. The variance inflation factor (VIF) tests the multicollinearity assumption (if VIF < 10, there is no multicollinearity). The suggested model’s regression hypothesis test is then run with the residuals assumed to have a normal distribution (histogram chart, normal P-P plot of regression standardized residual chart), and the linear connection is assumed (scatterplot chart).

The Ethics Committee in Biomedical Research of the Hue University of Medicine and Pharmacy approved the research proposal, Decision No. H2017/15. Written informed consent was obtained from all participants. The study protocol and experiments were performed in accordance with the Declaration of Helsinki.

## Results

Table 1 shows the general characteristics of the cohort. There was no statistically significant difference in the mother’s age or gestational age at birth between the disease and control groups. However, when considering the following characteristics, the difference between these two groups was statistically significant: umbilical artery pulse index, birth weight at delivery, mode of delivery, adverse pregnancy outcome, and UA pH.

**Table 1:** General characteristics of the study participants

Characteristics		Disease group (n_1_ = 135)	Control group (n_2_ = 135)	P
Mothers’ age (years)		30 ± 5.55	30 ± 5.54	1
Gestational age at birth (weeks)		35.17 ± 2.81	35.27 ± 2.97	0.78
Birth weight at delivery (gr)		1850 ± 454	2520 ± 713	< 0.001
Mode of delivery	Vaginal delivery	20 (14.8%)	59 (43.7%)	< 0.001
	Cesarean section	115 (85.2%)	76 (56.3%)	
	Assisted vaginal delivery	0	0	
Adverse Outcomes	No	42 (31.1%)	63 (46.7%)	< 0.001
	Yes	93 (68.9%)	72 (53.3%)	
UA pH	Mean	7.23 ± 0.03	7.26 ± 0.02	< 0.001
	< 7.20	34 (25.2%)	0 (0%)	< 0.001
	≥ 7.20	101 (74.8%)	135 (100%)	
UAPI		1.13 ± 0.33	0.90 ± 0.14	< 0.001

**Fig. 2:**
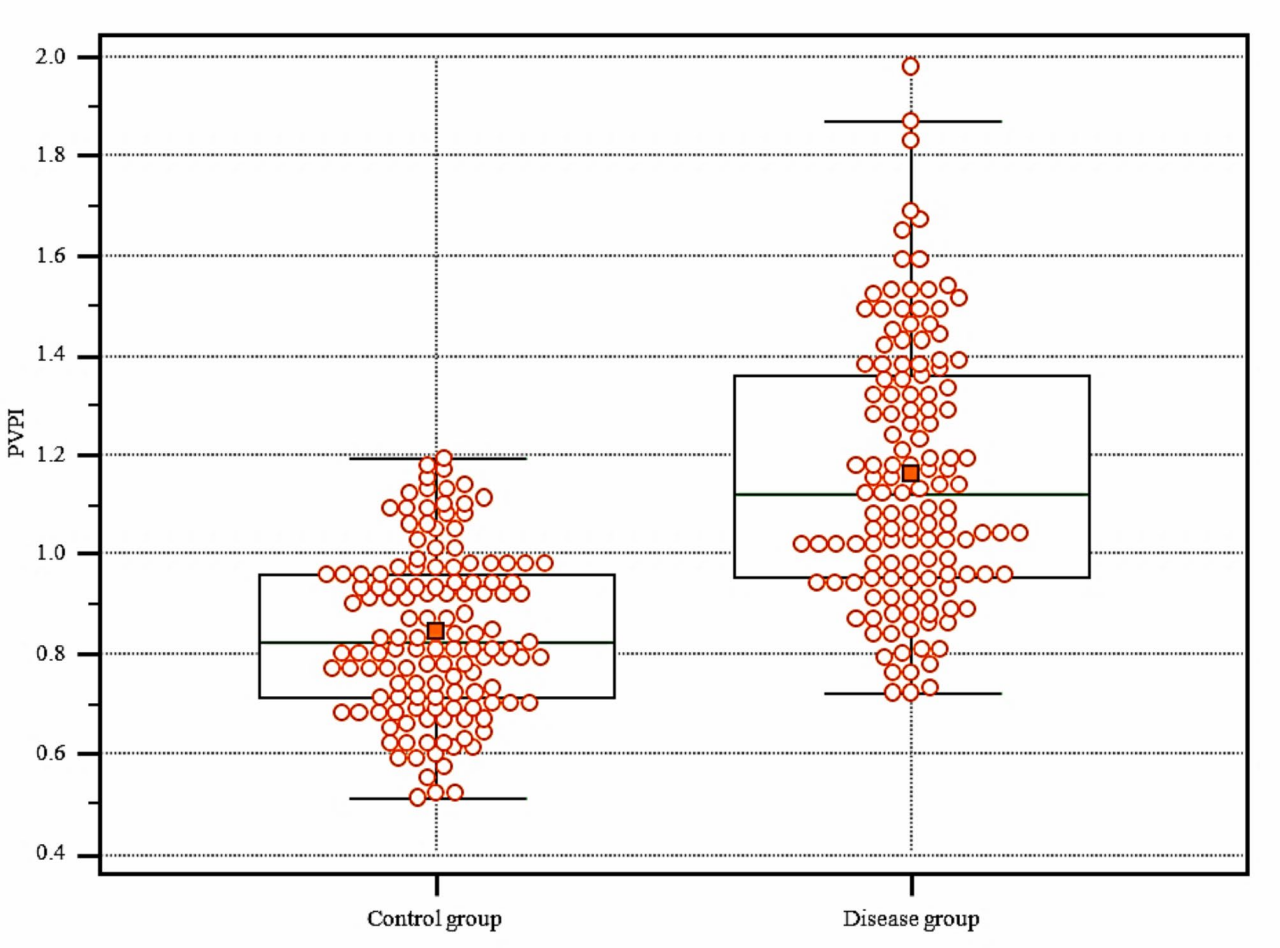
Distribution of the fetal PVPI of the FGR and normal groups

**Fig. 3:**
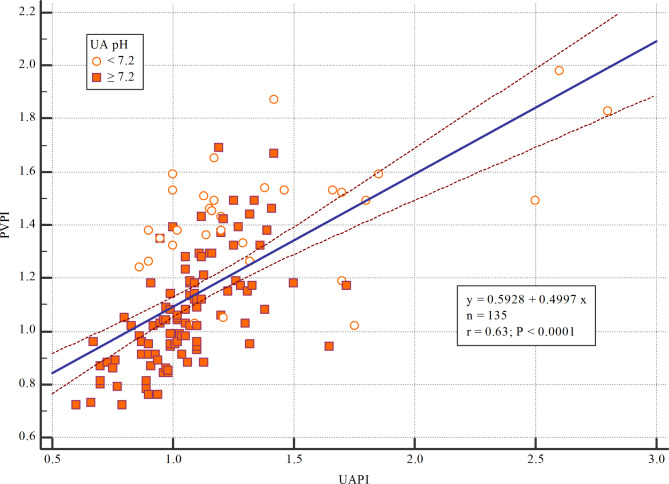
Correlation between PVPI and UAPI in the FGR group

**Fig. 4:**
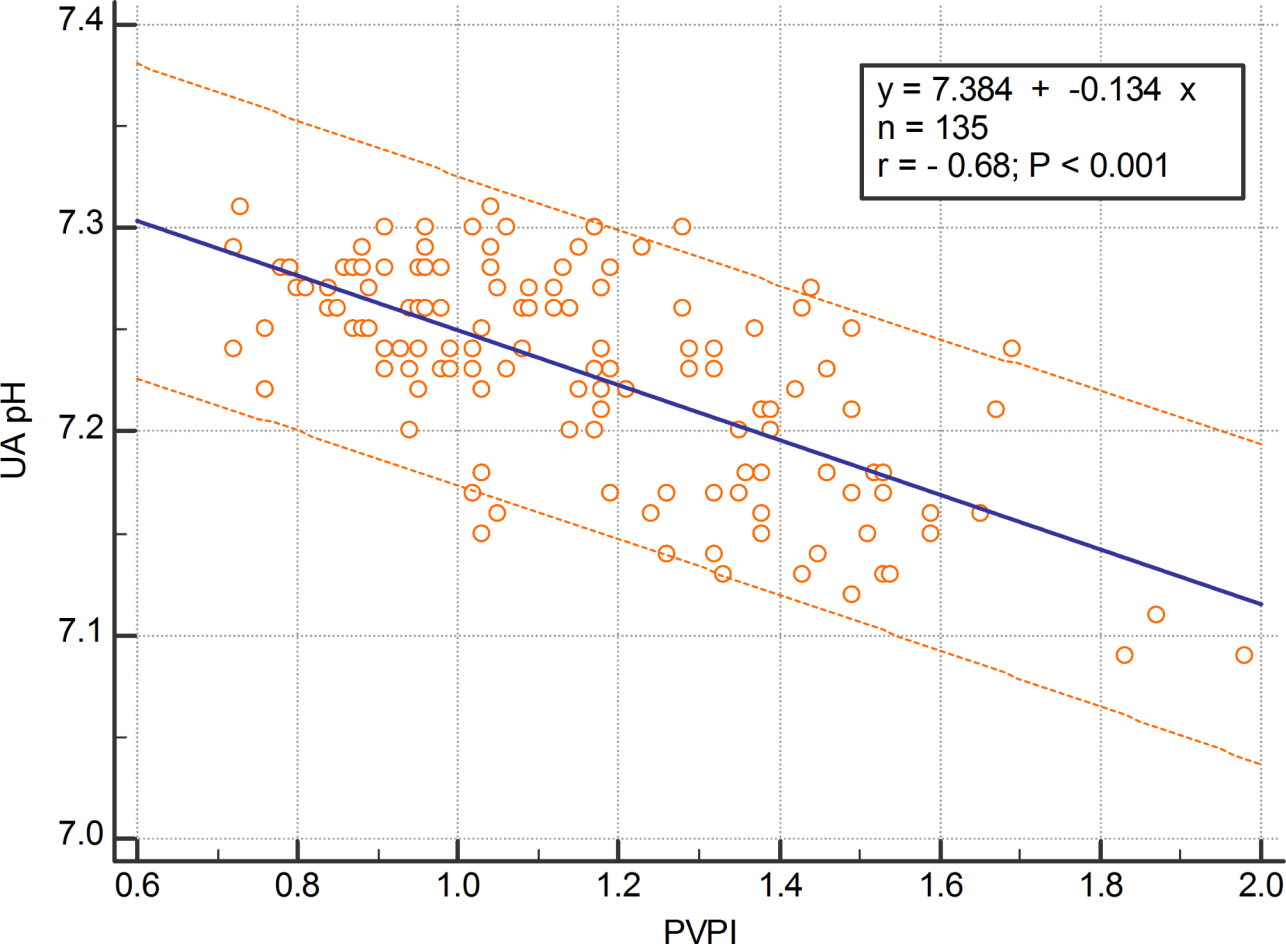
Correlation between PVPI and UA pH in the FGR group

**Fig. 5:**
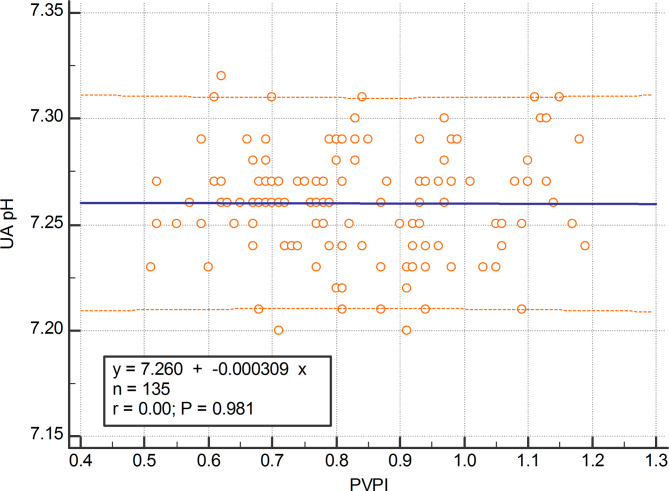
UA pH distribution according to PVPI in the normal growth group

The mean value of fetal PVPI was 0.84 ± 0.16 in the control group and 1.16 ± 0.26 in the disease group; the difference was statistically significant (p < 0.01) (shown in Fig. 2). In the FGR group, there was a positive correlation between UAPI and PVPI (r = 0.63; 95%CI: 0.52–0.72; p < 0.0001) (shown in Fig. 3). There was a negative correlation between UA pH and PVPI in the FGR group, with r = -0.68 (95%CI: -0.76 – -0.58); p < 0.001 (shown in Fig. 4). There was no correlation between UA pH and PVPI in normally growing fetuses (r = -0.002; p = 0.98) (shown in Fig. 5).

Table 2 depicts the distribution of cases in the fetal growth restriction group based on the umbilical artery (UA) pH cut-off point of 7.20 and fetal features. When umbilical artery blood pH was less than 7.20, the proportion of fetuses with adverse outcomes (excluding umbilical artery blood pH) increased to 91.2% in the growth restriction group, and this difference was statistically significant (p < 0.001). UA pH < 7.20 is a cut-off that increases the incidence of adverse pregnancy outcomes by 7.35 times (95% CI: 2.1–25.6; p < 0.001). A PVPI above the 95th percentile increased the likelihood of UA pH below 7.20 by 14.77 times (95% CI: 4.8–45.3; p 0.001).

**Table 2:** Distribution of cases according to umbilical artery (UA) pH cut-off in the fetal growth restriction group

Characteristics		UA pH < 7.20	UA pH ≥ 7.20	P	OR	95%CI
Adverse outcomes (UA pH excluded)	No	3 (8.8%)	42 (41.6%)	< 0.001	7.35	2.1–25.6
	Yes	31 (91.2%)	59 (58.4%)			
UAPI	> 95th percentile	23 (67.6%)	40 (39.6%)	< 0.005	3.18	1.40–7.25
	≤ 95th percentile	11 (32.4%)	61 (60.4%)			
PVPI	> 95th percentile	30 (88.2%)	34 (33.7%)	< 0.001	14.77	4.8–45.3
	≤ 95th percentile	4 (11.8%)	67 (66.3%)			
PVPI > 95th percentile and UA PI > 95th percentile	No	15 (44.1%)	76 (75.2%)	0.001	3.85	1.70–8.69
	Yes	19 (55.9%)	25 (24.8%)			

Based on a distribution of the number of fetuses with PVPI at the 95th percentile and UA pH at the 7.20 cut-off threshold, the diagnostic validity of PVPI was as follows: sensitivity: 88.2% (95%CI: 72.5 − 96.7%); specificity: 66.3% (95%CI: 56.2 − 75.4%); positive likelihood ratio: 2.62 (95%CI: 1.94–3.53 ); negative likelihood ratio: 0.18 (95%CI: 0.07–0.45 ); AUC: 0.77 (95%CI: 0.69–0.84); positive predictive value: 46.9% (95%CI: 39.5 – 54.3%); negative predictive value: 94.3% (95%CI: 86.8 − 97.7%).

## Discussions

Our matched cohort study demonstrated a significant change in PVPI in FGR cases and helped confirm the correlation between PVPI and UA pH in fetuses with FGR. FGR cases account for 3–7% of all births, depending on each country’s race and population characteristics [16]. In the past three years, many professional organizations such as International Society of Ultrasound in Obstetrics and Gynecology (ISUOG), Society for Maternal-Fetal Medicine, and American College of Obstetricians and Gynecologists (ACOG) have established guidelines for the diagnosis and management of FGR [17–19]. Evaluation of fetal growth should be an essential goal of antenatal care. 30% of stillbirths in the late third trimester are related to fetal growth restriction or small fetuses of gestational age. Thus, recognizing FGR is a crucial factor that needs to be included in strategies to reduce stillbirth rates [17]. The critical elements in the flowchart for diagnosing and managing FGR include ultrasound weight estimation, evaluation of fetal vascular Doppler features, and routine and computerized CTG [17, 18]. However, the role of pulmonary Doppler has not been addressed in the practice guidelines of medical associations, possibly because of the lack of previous case-control studies of pulmonary Doppler in FGR.

Some studies have demonstrated that in FGR cases, the fetal heart is likely to be restructured and could lead to later pathologies [20, 21], such as left ventricular hypertrophy that occurs in response to fetal hypoxia [22], and results in an alteration of fetal left ventricular dilatation and an increase in fetal left atrial pressure. The fetal pulmonary circulatory system consists of a wide distribution of pulmonary capillaries combined with a highly dilated mini-vein system to compensate for endogenous intrathoracic pressure as the fetus breathes and pressure from the atria. Thus, the pulmonary venous system completely rejects pulsatility from the fetal pulmonary artery and creates only mild pressure at the capillary ends of the veins. Therefore, PVPI is mainly due to the pulsatility pressure generated by the left atrium itself when the heart contracts [23]. In addition, there is increased flow through the patent foramen ovale into the left atrium to favor circulation to the fetal heart and brain. The consequence of this pathogenesis is an increase in PVPI in patients with FGR. The results of our study demonstrated that the mean value of fetal PVPI in the disease group (1.16 ± 0.26) was significantly higher than that in the control group (0.84 ± 0.16) (p < 0.001) (shown in Fig. 2). The study by Bravo-Valenzuela et al. also demonstrated that the PVPI in the FGR group (1.27 ± 0.39) was higher than that in the expected growth group (0.75 ± 0.12) (p < 0.001) [12]. A study by Lee et al. demonstrated that PVPI in the third trimester of pregnancy had a predictive value in fetuses that were smaller compared to the gestational age. PVPI exhibited a sensitivity of 70.27% and a specificity of 92.39% with a cut-off point of 1.13 for small-for-gestational-age pregnancy [6].

An association was observed between FGR, increased mortality or morbidity during the perinatal period, and subsequent adverse neurological outcomes. This suggests that fetal hypoxia or acidosis during FGR affects the preterm or full-term fetus [24]. Therefore, the measurement of UA pH is significant because it provides evidence for formulating recommendations in clinical practice. However, direct measurement of UA pH during pregnancy requires cord blood collection and increases the risk of complications from invasive procedures. Therefore, the application of noninvasive methods is recommended in clinical practice. For managing FGR in pregnancy, vascular flow Doppler has been increasingly indicated in routine practice and produces a reliable case-control dataset. A study by Figueras et al. demonstrated that in FGR cases, there was a negative correlation between UAPI and UA pH with r = -0.55 [8]. Suekane et al. reported a negative correlation between the pulsatility index of the ductus venosus and UA pH (r = -0.677) [7]. In this study, we found a positive correlation between UAPI and PVPI in the FGR group (r = 0.63, 95%CI: 0.52–0.72, p < 0.0001; shown in Fig. 3). Previous studies have discovered a moderately positive correlation between the Ductus venosus pulsatility index and the fetal PVPI [25]. That is why the hypothesis of a negative correlation between PVPI and UA pH in FGR cases is reasonable. Indeed, this study has demonstrated a negative correlation between PVPI and UA pH with r = -0.68 (95%CI -0.76 – -0.58; p < 0.001) (show in Fig. 4). The research findings (Table 2) clearly illustrate the relationship between UAPI, PVPI, and UA pH. The findings of this research demonstrate that PVPI and UAPI have a positive correlation in FGR and in the group of AU pH < 7.20, which includes 23 cases of fetuses with UAPI above the 95th percentile, 19 of these cases had PVPI above the 95th percentile. The likelihood of UA pH < 7.20 in FGR is therefore increased if the fetus has Doppler deterioration in both the umbilical artery and the pulmonary vein, with an OR rising from 3.18 to 3.85 (Table 2). This demonstrates PVPI’s utility in strengthening UAPI for predicting UA pH < 7.20.

Our study showed no correlation between PVPI and UA pH in the normal growth group (shown in Fig. 5), which could be related to the crucial vascular features that have the potential to decompensate and dilate blood vessels in general. Because of the different structures between veins and arteries, the decompensation capacity of veins is approximately eight times greater than that of arteries [26]. This suggests that the venous system can store a significant volume of blood while just slightly increasing pressure, showing a minor increase in vascular impedance and pulsatility. The pulmonary veins have a decompensation capacity similar to systemic veins; therefore, the pulsatility index does not exceed the normal range before significant hemodynamic changes occur. Under normal fetal growth conditions, the ability of the pulmonary veins to decompensate and dilate is still responsive to small hemodynamic changes. As a result, there is no correlation between UA pH and PVPI.

James et al. published an umbilical cord blood gas analysis in 1958, demonstrating that the fetus had a pre-existing hypoxic state [27]. Currently, cord blood gas analysis is a postpartum practice recommended by the Royal College of Obstetricians and Gynecologists and the ACOG for all high-risk pregnancies [28, 29]. In addition, some countries have adopted this practice as a routine process for all births. The practice of cord blood gas analysis is increasingly being adopted and serves as a source of evidence to interpret cord blood gas results with poor neonatal outcomes. Simultaneously, cord blood gas analysis will contribute to perfecting the legal basis for litigation related to obstetric complications. In this study, the mean UA pH of normally growing fetuses (7.26 ± 0.03) was significantly higher than that of the FGR group (7.23 ± 0.05) (p < 0.001) (shown in Table 1). Yeh et al. concluded that considering the association with adverse infant outcomes, the ideal UA pH was 7.26–7.30 [30]. Figueras et al. studied 117 fetuses with weight below the 10th percentile for gestational age, reporting that the UA pH had an average value of 7.25 ± 0.14 [8]. The sample criteria of Figueras et al.‘s study did not require the UAPI to be above the 95th percentile or the CPR to be below the 5th percentile. According to ISUOG, the fetuses in Figueras’s study sample would include FGR and small-for-gestational-age pregnancy cases. Therefore, the average UA pH value in Figueras’ study was higher than ours. However, this difference was not statistically significant (P = 0.12).

Goldaber et al. reported an association between UA pH < 7.20 and adverse neurological manifestations when studying 3506 full-term singleton pregnancies [30]. Casey et al. also found that infants born with UA pH < 7.20 persisting two h after birth had an adverse outcome than those with acidosis but recovered to normal UA pH levels [31]. Our study found that UA pH < 7.20 is a risk factor that increases the likelihood ratio of adverse pregnancy outcomes (OR = 7.35; 95% CI: 2.1–25.6, p < 0.001) (shown in Table 2). Furthermore, PVPI can predict fetal UA pH < 7.20 among FGR cases, with a sensitivity: 88.2%; specificity: 66%; positive likelihood ratio: 2.62 (95%CI: 1.94–3.53 ); negative likelihood ratio: 0.18 (95%CI: 0.07–0.45 ); AUC: 0.77 (95%CI: 0.69–0.84); positive predictive value: 46.9% (95%CI: 39.5–54.3%); negative predictive value: 94.3% (95%CI: 86.8–97.7%). To our knowledge, this is the first study to evaluate the possible implication of PVPI for clinical practice in predicting UA pH in FGR. Previous studies focused only on the value of umbilical venous Doppler [32, 33]. Rizzo et al. found that umbilical vein blood flow normalized for fetal abdominal circumference age was an independent predictor of adverse perinatal outcomes, including UA pH 7.10, with an AUC of 0.72 (95% CI: 0.642–0.804) [34].

Because UA pH affects the prognosis of both short- and long-term outcomes, it is critical to consider this when evaluating pregnancy outcomes. Table 3 shows the essential role of CTG and UAPI in estimating the UA pH of FGR using multiple linear regression. According to current FGR management guidelines, CTG and umbilical artery flow Doppler are critical factors in the decision to terminate a pregnancy [17, 18, 35]. As a consequence, the findings of this research support the importance of CTG and UAPI in FGR monitoring. Furthermore, we discovered an elevated risk of UA pH below 7.20 when PVPI was higher than the 95th percentile and the significance of PVPI in assessing UA pH in FGR. Although PVPI has a lower influence on UA pH than CTG and UAPI, it is an independent factor that affects UA pH (shown inTab.3).

**Table 3:** Independent variable coefficients in multiple linear regression models for estimating umbilical artery pH

Model	Independent Variable	Unstandardized Cofficients	Standardized Cofficients	t	Sig	Collinearity Statistics
		B	Beta			VIF
CTG	CTG	-0.048	-0.770	-13.90	0.000	1.00
UAPI	UAPI	-0.090	-0.554	− 7.67	0.000	1.00
CTG -UAPI	CTG UAPI	-0.043 -0.069	-0.689 -0.421	-15.95 − 9.76	0.000 0.000	1.03 1.03
CTG -UAPI - PVPI	CTG UAPI PVPI	-0.039 -0.053 -0.035	-0.621 -0.328 -0.176	-12.44 -8.65 -1.90	0.000 0.000 0.003	1.33 1.61 2.03
CTG -UAPI - PVPI - Mode delivery	CTG UAPI PVPI Mode delivery	-0.038 -0.053 -0.035 -0.002	− 0.618 − 0.329 − 0.175 − 0.016	-12.82 − 6.27 − 2.96 − 0.36	0.000 0.000 0.000 0.713	1.36 1.61 2.04 1.04

CTG: cardiotocography; UAPI: umbilical artery pulsatility index; PVPI: pulmonary vein pulsatility index

The data in Table S1 (Supplementary file) show that including PVPI in a multiple linear regression model containing CTG and UAPI enhanced the accuracy of the UA pH estimate. Since in various linear regression models that rely on variables such as UAPI, CTG, PVPI, and mode delivery to predict UA pH, the combination of CTG, UAPI, and PVPI produces the best-fit model because the adjusted R-squared of this model is the highest when compared to the other models. The Durbin-Watson value for this model is 1.73, suggesting that the findings do not contradict the first-order series autocorrelation assumption. The multiple linear regression model with the independent variables CTG, UAPI, and PVPI fits the population with a significant value equal to 0.000 in the ANOVA test. Furthermore, the suggested novel model has no multicollinearity by VIF values below 10. The impact of the mode of delivery on assessing the umbilical artery blood pH value of growth-restricted fetuses was not observed in this research since the coefficient of this variable in the regression model was not significantly statical (sig. = 0.71) (shown in Table 3), which numerous factors might cause. To begin with, the Cesarean section rate in this research is relatively high owing to indicators from the mother’s or the fetus’s conditions. Moreover, as advised by FIGO [35], the preferred method of delivery is elective Cesarean section if FGR protects the fetus from stress created by uterine contractions. As a result, in multivariable regression analysis, the mode of delivery did not affect UA pH. The histogram chart, the normal P-P plot of Regression Standardized Residual chart, and the scatterplot chart of this suggested model’s regression indicate the residuals assumed to have a normal distribution and the linear connection assumed. As a result, PVPI is a possible factor in predicting adverse pregnancy outcomes that should be studied further with larger sample sizes.

Based on our literature search, this could be the first study to assess PVPI values and establish their correlation with the prognostic validity of UA pH in FGR. Previous studies have concentrated solely on establishing a reference value range and analyzing the change in PVPI under several conditions. The attributes of this study demonstrated a positive correlation between UAPI and PVPI, and fetal PVPI may be utilized to predict UA pH.

Limitations of the research were that it did not investigate whether PVPI of the fetus’s right and left pulmonary veins differ in predicting fetal umbilical artery blood pH. Furthermore, the cohort was based on only one center. Moreover, this investigation did not study the effects of PVPI on UA pH in the early-onset or late-onset FGR groups. The difficulty in determining the precise onset time of FGR and insufficient fetuses having FGR before 32 weeks of gestation are two probable sources of this constraint. Lastly, we have yet to explore the effect of PVPI on fetal growth limitation induced by a placental malfunction. This topic is of great value, and further investigation is required to establish the clinical prognostic value of PVPI.

## Conclusions

In pregnancies with FGR, the increase in PVPI was statistically significant compared to that in the appropriate-growth group. In FGR cases, PVPI was positively correlated with UAPI and negatively correlated with UA pH. At the same time, fetal PVPI had a prognostic value of UA pH < 7.20. PVPI is a Doppler measurement that, like CTG and UAPI, should be investigated for further useful predictors of adverse pregnancy outcomes and proposed criteria for managing fetal growth restriction.

### Electronic supplementary material

Below is the link to the electronic supplementary material.

**Supplementary Material 1**

**Supplementary Material 2: Table S1**. Model fit comparison for determining umbilical artery pH by multiple linear regression.

## Data Availability

All data generated or analysed during this study are included in this published article and its supplementary information files.
