# Supplementary material for: Added value of the pulmonary vein pulsatility index and its correlation to neonatal umbilical artery pH in fetal growth restrictions: a Vietnamese matched cohort study
Source: BMC Pregnancy Childbirth. 2023 Aug 30;23:625. doi: 10.1186/s12884-023-05910-0 (PMC10468852; doi:10.1186/s12884-023-05910-0)
Supplement: Supplementary file 2 — Supplementary Material 2: Table S1. Model fit comparison for determining umbilical artery pH by multiple linear regression. [file 12884_2023_5910_MOESM2_ESM.docx]

**Supplementary files**

*Table S1. Model fit comparison for determining umbilical artery pH by multiple linear regression.*

| Model | Model Summary | | | ANNOVA |
| --- | --- | --- | --- | --- |
|  | R Square | Adjusted R Square | Durbin-Watson Statistic | Sig. |
| UAPI | 0.307 | 0.302 | 2.36 | 0.000 |
| CTG | 0.592 | 0.589 | 1.87 | 0.000 |
| CTG – UAPI | 0.763 | 0.760 | 1.80 | 0.000 |
| CTG – UAPI – PVPI | 0.779 | 0.774 | 1.73 | 0.000 |
| CTG – UAPI – PVPI – Mode of delivery | 0.779 | 0.772 | 1.73 | 0.000 |

CTG: cardiotocography, UAPI: umbilical artery pulsatility index, PVPI: pulmonary vein pulsatility index.
